# Gastrointestinal parasites of dogs (*Canis familiaris*) in Maiduguri, Borno State, Northeastern Nigeria: Risk factors and zoonotic implications for human health

**DOI:** 10.14202/vetworld.2019.1150-1153

**Published:** 2019-07-28

**Authors:** Kingsley Uwakwe Ezema, Samson Anjikwi Malgwi, Mohammed Kyari Zango, Falmata Kyari, Salamatu Mohammed Tukur, Ali Mohammed, Babagana Kachalla Kayeri

**Affiliations:** 1 Veterinary Teaching Hospital, University of Maiduguri, Borno State, Nigeria; 2 Department of Veterinary Parasitology and Entomology, Faculty of Veterinary Medicine, University of Maiduguri, Borno State, Nigeria

**Keywords:** coprological examination, gastrointestinal parasites, prevalence, zoonoses

## Abstract

**Aim:**

This study was designed to investigate the prevalence, associated risk factors, and zoonotic implications of gastrointestinal parasites of dogs in Maiduguri, Borno State, Northeastern Nigeria.

**Materials and Methods:**

A total of 200 rectal fecal samples were collected from dogs in Maiduguri for coprological examination using the saturated sodium chloride floatation technique. Detection of eggs or oocyst was done on the basis of keys of identification of parasites based on the morphology and size of eggs or oocyst.

**Results:**

The prevalence of gastrointestinal parasites of dogs was 31.5% (63/200) in Maiduguri. The prevalence of the infection was higher in young, male dogs kept outdoor in terms of age, sex, and management; the difference was statistically significant (p<0.05). The parasites detected in Maiduguri include *Ancylostoma* spp., *Toxocara* spp., *Dipylidium* spp., *Isospora* spp., and *Taenia* spp., with *Ancylostoma* spp. (16%) having the highest prevalence rate.

**Conclusion:**

Gastrointestinal parasites have high prevalence in Maiduguri and constitute potential risk to human health because all genera of parasites detected in the study area are of public health importance.

## Introduction

Dogs (*Canis familiaris*) have a close association with humans, providing security, companionship, and dietary protein requirement [1]. Dogs have been associated with more than 60 zoonotic diseases [2]. Helminthosis reportedly takes significant veterinary and public health importance worldwide [3]. Different types of enteric parasites have been reported, but *Ancylostoma caninum*, *Toxocara canis*, *Dipylidium caninum*, *Trichuris vulpis*, and *Echinococcus* spp. are the most common [4]. These infections exert serious health challenges in dogs and a variety of clinical signs such as unthriftiness, malaise, irritability, mild diarrhea, melena, vomiting, anorexia, anemia, and poor hair coat, ranging from the type of infection and density of the parasite. However, the infection may be asymptomatic [5].

Dogs are the most common pet animals worldwide, with several reports incriminating dogs as host of various intestinal parasites of zoonotic importance. These infections are transmitted to humans through direct contact with infected dogs or exposure to environments contaminated with infected dog feces; alternatively, the larvae can penetrate the skin of the susceptible host [6]. Other factors that also play a key role in the transmission of these infections to humans include wind, rain, arthropods, human, and vehicular traffic, which can aid the spread of the infective stages of parasites present in dog feces to human food and water sources. Children are most susceptible or at higher risk of exposure to the infection. Human infection with *Toxocara* spp. is asymptomatic, although some individuals may develop visceral larval migrans and ocular toxocariasis. *Ancylostoma* spp. have been reported as an etiological agent for cutaneous larval migrans and are associated with eosinophilic enteritis in humans [7].

In Nigeria, several reports exist on the endemicity of the infection in various states of the country, across various geographical distributions [8-10]. However, there is a paucity of information on the prevalence, risk factor, and public health significance of zoonotic infection to the human population especially when there is an increase in dog population for security, hunting, and breeding purposes, especially in developing nations.

This study aimed to provide information on gastrointestinal parasites of dogs and their risk factors and zoonotic implications to human health in Maiduguri. Data provided by this study will be utilized by practicing veterinarians who will be aware of parasites in their environment with its zoonotic significance which will guide their advice to dog owners, highlighting the dangers of these parasites and providing them with preventive measures which will, in turn, reduce the risk of zoonotic transmission.

## Materials and Methods

### Ethical approval

All samples were collected using standard sample collection without inflicting pain or harming the animals. The research was conducted after the approval of the Research and Ethics committee of University of Maiduguri, Nigeria.

### Study area

This study was conducted in Maiduguri, the capital of Borno State, which lies in the Northeastern geopolitical zone. The state is situated within the semi-arid zone of West Africa. It lies on latitude 11^o^ 5`N and 13^o^5`E. It has a total area of 72,609 square km. It is bordered by the Republic of Niger to north, Cameroon Republic to east, and Chad to northeast regions. The temperature ranges from 35°C to 40°C for most of the year. It is characterized by two distinct seasons: A short rainy season (June-September) and a long dry season (October-June) [11].

### Sample collection

A total of 200 dogs were sampled in the study area; sampling was done based on convenience and prior to sample collection, owners’ consent was obtained. A cross-sectional study was conducted from March to October 2018 on dogs with no bias to any of the risk factors. The dogs were properly restrained. About 5-10 g fecal samples were directly collected from the rectum using one of the gloved hands, while the other hand was used to support the caudoventral abdominal area, and the fecal samples were transferred to plastic containers. These containers were properly labeled with the age, sex, breed, and the management practice of the dogs. These samples were transported in an ice pack to the Department of Veterinary Parasitology and Entomology, University of Maiduguri, Borno State, Nigeria, for laboratory analysis.

### Laboratory analysis

#### Floatation technique

About 2 g of feces was put into a universal bottle, and 5 ml of floatation medium was added; in this study, the floatation medium was saturated salt solution with a specific gravity of 1.20. The sample was broken and properly mixed with the floatation medium using a glass rod. It was sieved into a centrifuge tube and later placed on a tube rack. More of the medium was added until a convex meniscus was formed. A coverslip was placed on the filled tube and left to stand for 3-5 min. The coverslip was later placed on a glass slide and examined microscopically for eggs or oocysts. The eggs/oocysts were classified up to the genera level by keys for their identification based on their morphology and characteristics [5,12]. Each sample was categorized as positive if at least one egg or an oocyst was observed [13] by microscopy in the employed technique.

### Statistical analysis

The prevalence was calculated for all data as the number of infected individuals divided by the number of examined individuals, and was expressed in percentage by multiplying by 100. Chi-square test was employed to determine the statistical association between age, sex, breed, and management practice of dogs using GraphPad Prism 5.0 (San Diego, USA).

## Results

Table-1 summarizes the overall prevalence of 31.5% in the study area and the prevalence of the infection based on associated risk factors. In terms of age, there was higher prevalence of the infection in the young (50.7%) as compared to the adults (20.9), which was statistically significant. There was also a higher prevalence in males (43.3%) than females (27.0), which was also statistically significant. Crossbreed dogs had a higher prevalence rate of 42.1% among both exotic and local breeds with 19.6% and 35.2%, respectively. In terms of management, dogs that kept outdoor (53.8%) had a higher prevalence rate as compared to those managed indoor (12.8%); the difference was statistically different.

**Table 1:** Prevalence and associated risk factors of gastrointestinal parasites of dogs (*Canis familiaris*) in Maiduguri, Borno State, Northeastern Nigeria.

Risk factors	Number of examined dogs	Number of infected dogs	Prevalence (%)
Age			
Young (< 1year)	71	36	50.7^a^
Adult (> 1year)	129	27	20.9^b^
Total	200	63	31.5
Sex			
Male	83	36	43.3^a^
Female	117	27	27.0^b^
Total	200	63	31.5
Breed			
Exotic	56	11	19.6^a^
Cross	19	08	42.1^a^
Local	125	44	35.2^a^
Total	200	63	31.5
Management			
Owned (indoor)	109	14	12.8^a^
Stray (outdoor)	91	49	53.8^b^
Total	200	63	31.5

Number with same superscript in the 3^rd^ column did not differ statistically significantly (p>0.05)

The prevalence of various genera of gastrointestinal infection in dogs is presented in Table-2. Six different genera of gastrointestinal parasites were observed in the study which include *Ancylostoma* spp. (16%), *Toxocara* spp. (7.0%), *Dipylidium* spp. (4.5%), *Isospora* spp. (2.5%), *Taenia* spp. (1.5%), and *Ancylostoma* spp.

**Table 2:** Prevalence of gastrointestinal infection by genera in dogs (*Canis familiaris*) in Maiduguri, Borno State, Northeastern Nigeria.

Parasites	Number of positive samples	Prevalence (%)
*Ancylostoma* spp.	32	16
*Toxocara* spp.	14	7.0
*Dipylidium* spp.	9	4.5
*Isospora* spp.	5	2.5
*Taenia* spp.	3	1.5

## Discussion

This study revealed an overall prevalence of 31.5% of gastrointestinal parasitic infection in dogs from Maiduguri, Northeastern Nigeria. This study is in consonance with the findings in rural communities in Central Nigeria, [14] in Maiduguri [15], and in Venezuela [16], reported prevalence rates of 37.3%, 38%, and 34.8%, respectively. However, the prevalence rate in this study is lower than the reports from Markudi [17], Zaria, [18] and Kaduna [19], reported prevalence rates of 73.7%, 62.7%, and 93.8%, respectively. These variations in the prevalence rate could possibly be due to sampling size, geographical variations, season of the study, access to veterinary services, the presence or absence of intermediate host, sampling protocols, demographic factors, and diagnostic techniques. Another key factor is the state of health of the dogs sampled in the study; for instance, Ogunkoya *et al*. [18] focused on clinically sick dogs presented to a veterinary teaching hospital, which were most times diseased. The result of the study showed a higher prevalence rate of infection in the young (50.7%) compared to the adults (20.9%) where the difference was statistically significant (p<0.05). This agrees with the report of Mustapha *et al*. [15] but disagrees with the findings of Ehimiyein *et al*. [20] who reported a higher prevalence in adults. This could be associated to the fact that adults who are mostly carriers shed infective eggs after acquiring immunity from previous exposure of the infection; it is also a known fact that there is a possibility of transmission of helminths through the placenta or milk [5].

A higher prevalence rate of infection was observed in males (43.3%) compared to females (27.0%), which was statistically significant (p<0.05). This is in consonance with the report of Mustapha *et al*. [15] and disagrees with the findings of Ehimiyein *et al*. [20] who reported a higher prevalence of the infection in female dogs. The high prevalence of the infection in males could be attributed to the fact the male dogs travel long distances, especially during breeding season in search of mates. Dogs kept indoors had a lower prevalence rate compared to those kept outdoor, which was statistically significant (p<0.05), due to the tendency of the dogs to be exposed to natural infections, poor management of animal health, and high degree of environmental contamination. [17]. In terms of breed of the dogs, crossbreed dogs had the highest prevalence of infection; however, the difference was not statistically significant (p>0.05).

The gastrointestinal parasites observed in this study were *Ancylostoma* spp., *Toxocara* spp., *Dipylidium* spp., *Isospora* spp., and *Taenia* spp. These parasites were similar to the reports from Maiduguri [15], Zaria [18], Kaduna Metropolis [19], and Zaria [20]. In this study, *Ancylostoma* spp. was the most prevalent species to cause infection, which is in consonance with the report of Mustapha *et al*. [15] but disagrees with the findings of Ehimiyein *et al*. [20] who reported *Taenia* spp. as the most prevalent species to cause infection. The higher prevalence of *Ancylostoma* spp. among all the other parasites suggests that the parasite is highly infective and efficiently transmitted; the ovum containing this species is maintained after being expelled in dog feces for a long period of time. This finding, therefore, highlights the importance of preventive measures because this parasite is of serious zoonotic implication causing eosinophilic enteritis and cutaneous larva migrans in humans. The high prevalence of *Toxocara* spp. has been associated with the ability of the adult parasite to shed a large quantity of infective eggs into the environment. The parasite is infective to humans causing visceral and ocular migrans [4,6].

The results of this study clearly indicate that dog owners, especially their children, are at risk of the infection; this is because of their closeness to pets and the possibility of them to ingest sand.

## Conclusion

This study clearly shows the endemicity of gastrointestinal parasites in Maiduguri, Northeastern Nigeria. The general public, especially dog owners and those who consume dog meat, fall within the high-risk category of acquiring the infection. Public health enlightenment campaigns by veterinarians with key focus on preventive measures such as regular deworming are required.

## Authors’ Contributions

KUE and SAM were involved in designing the research and drafting the manuscript, while MKZ, FK, AM, SMT, and BKK were involved in laboratory procedures. All authors read and approved the final manuscript.
